# Older postmenopausal women with lower lean mass have hypermethylated sites in the PI3K-Akt pathway

**DOI:** 10.3389/fphys.2023.1150821

**Published:** 2023-04-10

**Authors:** Igor Massari Correia, Guilherme da Silva Rodrigues, Natália Yumi Noronha, Lígia Moriguchi Watanabe, Mariana Luciano de Almeida, Andressa Crystine da Silva Sobrinho, Carla Barbosa Nonino, Carlos Roberto Bueno Júnior

**Affiliations:** ^1^ School of Physical Education and Sport of Ribeirão Preto, University of São Paulo, São Paulo, Brazil; ^2^ Department of Internal Medicine, Ribeirão Preto Medical School, University of São Paulo, São Paulo, Brazil; ^3^ Department of Health Sciences, Ribeirão Preto Medical School, University of São Paulo, São Paulo, Brazil; ^4^ College of Nursing of Ribeirão Preto, University of São Paulo, São Paulo, Brazil

**Keywords:** epigenetic, DNA methylation, muscle loss, muscle strength, body composition

## Abstract

**Introduction:** The decrease in lean mass is directly related to the loss of independence, muscle strength, and worse quality of life over the years. Although the genetic determinants of muscle mass were well recognized, recent literature has been uncovering new epigenetic factors affecting the state of muscular tissue. This study aimed to verify differences in the DNA methylation profile among Brazilian postmenopausal women aged 50–70 years according to the lean mass evaluation.

**Methods:** A cross-sectional study comprised 40 women aged 50–70 years. After K-means cluster analysis the 40 participants were divided into two groups, the Lower Lean Mass group with 20 participants (61.1 ± 4.6 years) and the Higher Lean Mass group with 20 participants (60.7 ± 3.2 years). Lean mass was measured by dual-energy X-ray emission densitometry (DEXA). The participants' DNA was extracted using the Salting Out technique and subsequently, the Illumina 850k EPIC Infinium Methylation BeadChip was performed to obtain methylation data.

**Results:** We obtained 1,913 differentially methylated sites (*p* ≤ 0.005 of β > 5% and β < −5%) in a total of 979 genes between groups (*p* ≤ 0.005; −5% > β > 5%). In addition, the PI3K-Akt pathway had the greatest power of significance with an FDR of 4.6 × 10^–3^.

**Conclusion:** Our results demonstrate a differentiation between specific sites of different genes, which have essential functions in body composition and energy metabolism, supporting future studies that aim to relate lean mass with epigenetics.

## 1 Introduction

The aging process is one of the main predictors of chronic diseases related to the majority of morbidities, hospitalizations, health costs, and mortality, representing risk factors for geriatric syndromes, frailty, immobility, and decreased physical resilience. In parallel, in the aging process, a decrease in cell regeneration capacity and a simultaneous increase in cell apoptosis is related to the loss of cardiorespiratory function and a decrease in muscle mass and strength. Lean mass plays an important role in aging, as its decrease is directly associated with loss of independence, muscle strength, and lower quality of life over the years (Taekema et al., 2010).

Studies have been showing that reduced muscle mass levels can increase the risk of mortality in older adults by 11 times, and when associated with cardiovascular diseases, the risk of mortality increases by 14 times (Santana et al., 2019). Around age 40, a more pronounced decrease in lean mass is observed and can result in sarcopenia, which is defined as the involuntary loss of skeletal muscle mass and a decrease in strength related to age (Walston, 2012). The rate of muscle mass decrease, and consequently the development of sarcopenia, depends on the individual lifestyle, especially the physical activities practice, and diet (Beaudart et al., 2017). Sarcopenia is considered a health problem due to its consequences on muscle functionality and body composition. Currently, the annual health costs related to sarcopenia in the United States are estimated at 18 billion dollars, as lean mass reduction is associated with diseases such as obesity, diabetes, and osteoporosis (Janssen et al., 2004). In Brazil, the costs of the Unified Health System (SUS) were 3.45 billion reais in 2018 (more than 890 million dollars), of which 72% of the costs were for individuals between 30 and 69 years and 56% for women. The analysis of the World Economic Bank estimated that countries such as Brazil, China, India, and Russia lose more than 20 million productive years of life per year as a result of chronic non-communicable diseases (NCDs), generating losses in the Brazilian economy of US$ 4.18 billion between 2006 and 2015 (Malta et al., 2017).

Studies in epigenetics can provide precise answers regarding physiological responses at the molecular level. This can be applied to variations of human lean body mass in the aging process since older individuals are more prone to modifications in DNA methylation patterns that could affect the expression of genes related to lean mass regulation (Pal and Tyler, 2016). Hypermethylation, in most cases, leads to gene silencing, while hypomethylation could increase gene expression (Baylin and Jones, 2011; Messerschmidt et al., 2014). Thus, knowing the relations between methylation profiles according to lean mass could be a decision-making helper to health professionals to prevent the reduction and physiological alterations in the lean mass.

Indeed, there is evidence in the literature suggesting the influence of DNA methylation on the expression of metabolically essential genes in skeletal muscle and its possible implication in susceptibility to age-related metabolic diseases (Gensous et al., 2019). However, little is known about methylation patterns in lean mass in healthy older people (Gensous et al., 2019). One study compared the DNA methylation profile in the skeletal muscle of 24 older males with 24 young males. The authors identified 5,963 differentially methylated CpG sites between the two groups, and most of these (92%) were hypermethylated in the older group, suggesting epigenetic links between post-mitotic skeletal muscle aging and DNA methylation (Zykovich et al., 2014). In a longitudinal study carried out with 1,550 middle-aged female twins, the authors identified seven regions whose methylation status was significantly associated with the variation in skeletal muscle mass, demonstrating that epigenetic studies have an essential role in the study of muscle mass (Livshits et al., 2016).

It is important to emphasize the lack of studies comparing the DNA methylation profile in older people with different values of lean mass, and in postmenopausal women. Longitudinal studies have shown that in older women, the muscle mass rate loss is 0.64%–0.70% per year (Mitchell et al., 2012), and could be associated with menopause since the natural decline in estrogen increases visceral fat mass and decreases bone mass density, muscle mass, and strength (Maltais et al., 2009). Thus, the study aimed to verify differences in the DNA methylation profile among Brazilian postmenopausal women aged 50–70 years according to the lean mass evaluation. We hypothesize that women with a higher lean mass (53.2%–60.9%) have different methylation profiles from women with a lower lean mass (45.2%–52.2%).

## 2 Methods

The research project was submitted and approved by the Ethics Committee for Human Research of EEFERP-USP (CAAE: 79582817.0.0000.5659), based on Resolution 466/12 of the Ministry of Health, regulated by the National Health Council. This study was registered in the Brazilian Registry of Clinical Trials (REBEC) with the registration number RBR-3g38dx.

Women postmenopausal aged 50–70 years enrolled in the Physical Education Program for the Elderly (PEFI) of the School of Physical Education and Sport at USP de Ribeirão Preto (EEFERP-USP) were invited to participate in our study. It is noteworthy that the study participants were considered physically inactive through the Modified Baecke Questionnaire for the Elderly, with a score ≤9.11, 6 months before the beginning of the study (Rodrigues et al., 2023). A total of 40 women were eligible to participate in the study. After measuring lean mass by DEXA, the participants were divided into two groups: higher lean mass (HLM) and lower lean mass (LLM). The HLM group included 20 participants with the highest percentage of lean mass, and the LLM group included 20 participants with the lowest percentage of lean mass, measured by DEXA. When we separated into adults and older women, the group with the lowest mass had 10 adult women (55.8 ± 2.4 years with mean and 48.10% of lean mass) and 10 elderly women (64.8 ± 2.2 years with mean and 49.74% of lean mass). While the group with the highest lean mass consisted of 9 adult women (56.1 ± 2.7 years with an average of 57.95% of lean mass) and 11 elderly women (63.1 ± 1.5 years with an average of 55.49% of lean mass).

### 2.1 Physical and functional assessment instruments

Anthropometric assessments were performed to characterize the participants: body mass, height, body mass index (BMI), and waist circumference (WC) (Organization, 1995). The Food Consumption Markers Form (FMCA) of the Ministry of Health was used to assess the participants’ diet, and the modified Baecke Questionnaire for the elderly to assess their physical activity level (MBQE) (Mazo et al., 2001; Saúde, 2021).

The following motor tests from the Rikli and Jones battery of tests were performed: elbow flexion and extension (EFC) test to assess upper limb strength, sit-to-stand test (STS) to assess lower limb strength and 6-min walk (6MWT) to assess the participants’ aerobic fitness (Rikli and Jones, 2013). In addition, we performed the maximum repetitions test to complement the participants’ strength assessment, using the incline bench press and the 45° leg press for this purpose (Osness, 1990).

### 2.2 DEXA evaluation

A laboratory technician performed measurement and body composition analyses following standard procedures. At the time of the evaluations, the participants were not wearing any accessories, such as necklaces, bracelets, and earrings, to reduce errors in the results (Hansen et al., 1999; Chen et al., 2007). The DEXA scan analysis was performed blindly by the technician responsible for the analysis. After having all the reports, the researchers performed the cluster analysis to group the participants.

The whole-body scans were manually analyzed for total and percentage lean tissue mass and total and percentage fat mass (Hansen et al., 1999; Chen et al., 2007).

### 2.3 Blood tests

Blood tests for glucose, insulin, and glycated hemoglobin were performed after the participants had fasted for 12 h. The blood material was immediately sent to the Faculdade de Ciências Farmacêuticas da USP de Ribeirão Preto (FCFRP), wrapped in ice in a thermal box, and analyzed using the Wiener Lab BT 3000 plus autoanalyzer (Moraes et al., 2011). The reagents used for the analyses described in this topic were from the same batch (LABORLAB).

### 2.4 DNA extraction and methylation assay

A peripheral blood sample was collected in tubes with EDTA in a sterile environment, and DNA was extracted from leukocytes. Genomic DNA was isolated from 500 μL of peripheral blood by the *salting out* method (Freire et al., 2006). DNA concentration and quality were evaluated by spectrophotometry at 260 and 280 nm (A260 and A280) using Biodrop, and integrity was evaluated using 1% agarose gel - the run protocol adopted was 90 min at 80 W (Yoza et al., 2002). In addition, the extraction technique was evaluated using negative controls. Subsequently, the samples were stored at −20°C. Leukocyte genomic DNA (500 ng) was treated with bisulfite using the DNA EZ Methylation-Gold methylation kit (Zymo Research, CA, United States), following the recommendations stipulated by the manufacturer.

DNA methylation assays were performed using the *array* technique, with the Illumina EPIC Infinium Methylation BeadChip, following the manufacturer’s instructions (Guo et al., 2014; Stirzaker et al., 2017)*.* The Illumina iScan system detects Infinium BeadChip fluorescence, the intensities are exported as *raw data* using the chAMP package (for the R platform), and the data are converted into beta methylation values, processed, and analyzed (Tian et al., 2017; RStudio, 2019).

### 2.5 DNA methylation analysis

Methylation analyses were performed using RStudio software and calculated using the Bioconductor chAMP data package. The DMP function based on the limma algorithm (da Silva Rodrigues et al., 2023) was used to find the differentially methylated sites between the two groups, classified as hypermethylated and hypomethylated, which presented values of *p* ≤ 0.005 and β > 5% for hypermethylated sites and *p* ≤ 0.005 and β < −5% for hypomethylated sites. We also adopted the DMR function from the same package, which presents the differentially methylated regions, to analyze whether, in addition to DMPs, the difference in lean mass was also reflected in the DMRs.

### 2.6 Enrichment analysis

Enrichment was obtained through gene enrichment analysis (GSEA). This method is based on comparing a list of genes (LG), ordered according to their correlation with the phenotype, and the genes described in a specific metabolic pathway (MP) (Noronha et al., 2022). To this end, calculations of the enrichment index, significance levels, and corrected significance levels for multiple tests are performed (da Silva Rodrigues et al., 2023; Noronha et al., 2022). The GSEA evaluates each gene independently, ignoring the relationships between them. The ShinyGO v0.66: Gene Ontology Enrichment Analysis database was used to perform interaction and functional enrichment. We adopted an FDR <0.05 for the human organism to select the gene-related KEGG metabolic pathways to analyze the results (Ge et al., 2020).

### 2.7 Statistical analysis

Two researchers entered the characterization data of the participants and biochemical variables into Excel spreadsheets, and then the information was cross-referenced to eliminate possible typing errors. We used the Statistical Package for the Social Sciences (SPSS) software for statistical processing. We used the Shapiro Wilk as a normality test, and as the data presented parametric distribution, we adopted the Student’s t-test, *p* < 0.05.

The M value was adopted to compare the difference in methylation of the sites of the genes that showed statistical differences by DMR, which were selected and passed through the quality control of chAMP and Bumpunter (*p* ≤ 0.05). To use the M value, it was necessary to transform the beta values into the M value. This approach has been widely used in the analysis of DNA methylation microarrays, as it increases the range of values, thus better performing statistical analyses than the beta values. The beta values $\left(Beta\right)$ of the sites were converted into M values $\left(M_{i}\right)$ using the following equation on a logarithmic scale (Du et al., 2010; Tian et al., 2017):

$$M_{i}=\log_{2}\left(\frac{{Beta}_{i}}{1-Beta}\right)$$

Multiple linear regression was performed with the variables studied at the cross-sectional moment of the intervention. We used multiple linear regression to analyze interactions between lean mass and body mass index. In the multiple linear regression model, the stepwise forward input method was considered to calculate the adjusted estimate (β), with a 95% confidence interval (95% CI). The *R* ^2^ was analyzed to verify the percentage of determination of the coefficient of variation, which explains the model. The multiple linear regression analysis was performed considering the lean mass level as the dependent variable and the BMI as a predictor, it is noteworthy that the regression was adjusted for age. Data were analyzed using the statistical program Statistical Package for the Social Sciences (SPSS)^®^ version 20.0.

To separate the groups, we used the K-means clustering method, by classifying the participants into multiple groups, so that the intra-cluster variation is minimized by the sum of the squares of the Euclidean distances between the items and their centroids (WU, 2012).

## 3 Results

**FIGURE 1:**
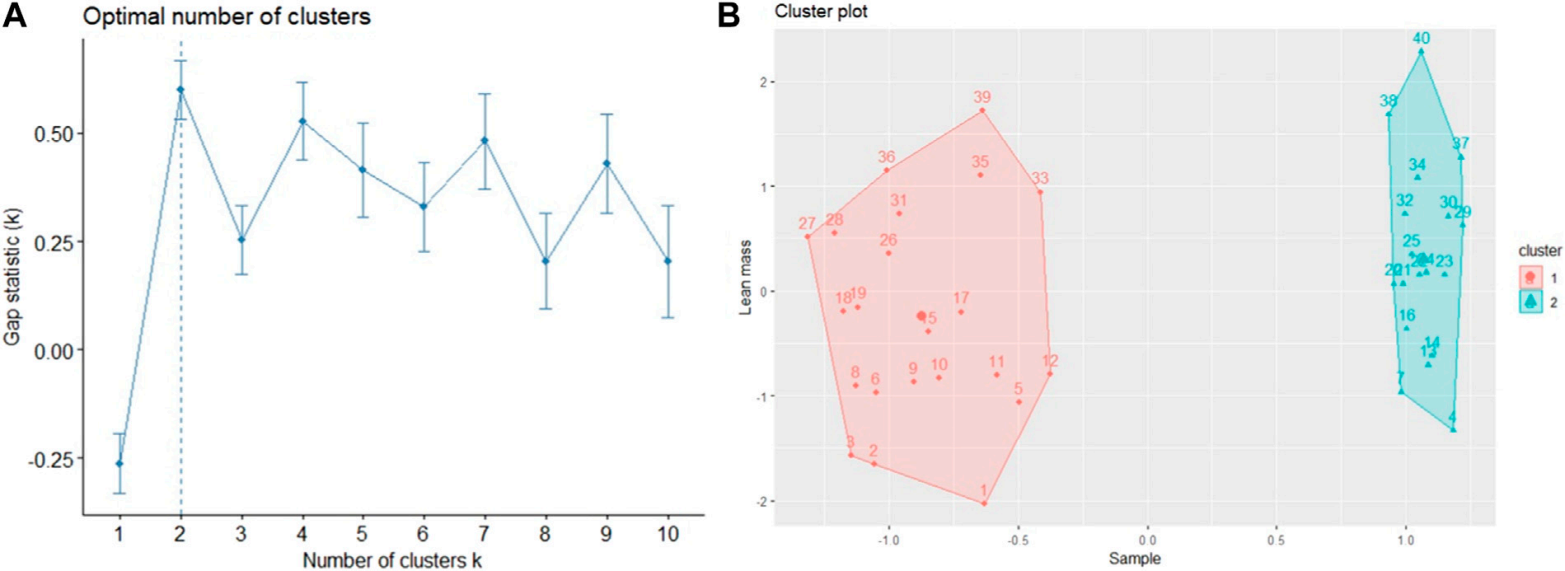
Participants grouped by lean mass values. Note: **(A)**- ideal number of groups using the lean mass variable; **(B)**- red distribution: group with lower lean mass; green distribution: group with greater lean mass.

After K-means analysis to identify the number of participants by the lean mass values (Figure 1A), we performed the division into two groups (Figure 1B) groups, the Lower Lean Mass group (LLM) with 20 participants (61.1 ± 4.6 years) and the Higher Lean Mass group (HLM) with 20 participants (60.7 ± 3.2 years).

There was a similarity between the groups in terms of age, motor, and functional tests (RM, STS, EFC, and 6MWT), waist circumference, and assessments of the participant’s level of physical activity and daily diet (MBQE and FMCA) (Table 1). Body composition measurements showed significant differences between groups (BMI and lean mass percentage - *p* < 0.05) (Table 1). Although participants in the HLM group presented significantly greater lean mass than participants in the LLM group (*p* < 0.001), there were no differences in performance in functional tests between groups.

**TABLE 1:** Anthropometric data and physical fitness profiles in women aged 50–70 years, separated by groups (LLM and MLM).

Variables	Lower lean Mass (*n* = 20)	Higher lean Mass (n = 20)	*p-value*
Lean Mass (%)	49.1 ± 2.5	56.7 ± 2.8^*^	<0.01
Age (years)	61.1 ± 5.3	60.5 ± 4.2	0.62
BMI (kg/m^2^)	31.7 ± 3.6	27.7 ± 3.4^*^	0.01
WC (cm)	96.9 ± 8.0	91.9 ± 12.2	0.14
EFC (reps)	14.9 ± 5.0	17.3 ± 4.3	0.10
RM supine (kg)	22.4 ± 4.4	22.1 ± 4.2	0.87
RM leg press (kg)	130.7 ± 28.4	123.9 ± 40.8	0.56
STS (reps)	22.1 ± 8.1	18.3 ± 8.0	0.15
6MWT (m)	538.2 ± 64.7	523.8 ± 59.3	0.46
MBQE (points)	5.8 ± 3.8	4.0 ± 2.8	0.11
FMCA (points)	17.3 ± 10.3	19.6 ± 8.7	0.47

Legend: BMI, body mass index; WC, waist circumference; EFC, elbow flexion and extension; RM, maximum repetition; SEL, sit and stand; 6MWT, 6-min walk test; BME, modified baecke questionnaire for the elderly; FMCA, food consumption markers form; cm, centimeters; m, meters; reps, repetitions; kg, kilos. *Student’s t-test, *p* < 0.05.

Multiple linear regression was used to generate the final model, which revealed an association between lean body mass and BMI. The model was adjusted for age and the overall model accounted for 43% (*R* ^2^ = 0.434) of the variation in muscle mass measurement in the BMI variable (Table 2).

**TABLE 2:** **Final model of the multiple linear regression analysis considering lean mass as the dependent variable, adjusting the model for age, and using BMI as a predictor**.

Variables	Estimates	CI 95%	*p*-valor	*R* ^2^
Years	−0.010	−0.257/0.237	0,93	0.001
BMI	−0.660	−1.031/−0.456	<0,01*	0.434

Legend: Estimates, Adjusted Beta; CI 95%, Confidence Interval, R2 proportion of variation in the dependent variable explained by the independent variables; BMI, body mass index.

**FIGURE 2:**
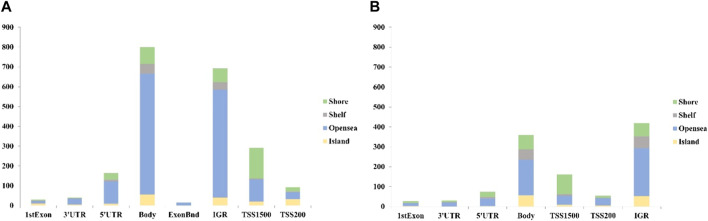
Distribution of hypermethylated and hypomethylated sites. Note: **(A)** The number of hypermethylated sites per gene region in the LLM group (1,776). **(B)** The number of hypomethylated sites per gene region in the LLM group (137). The X-axis of the graph shows the regions of genes and intergenic regions, and the Y-axis is the number of hypomethylated sites. Each color represents a region of the CpG islands related to hypomethylated genes.

After quality control with the chAMP package, we obtained 719,902 valid sites—of these 9,032 were significantly different between groups (*p* ≤ 0.005) and 1,913 were differentially methylated (*p* ≤ 0.005 of β > 5%, and β < −5%). Figure 2A shows the differentially hypermethylated sites in the LLM group compared to the HLM group, totaling 1,776 sites, distributed in 1stExon, 3′UTR, 5′UTR, Body, ExonBnd, TSS1500, TSS200, and IGR. We notice that the open sea CpG islands, the Body, and IGR had the highest hypermethylation frequency. In Figure 2B, 137 sites hypomethylated sites were distributed in the body and the IGR.

**FIGURE 3:**
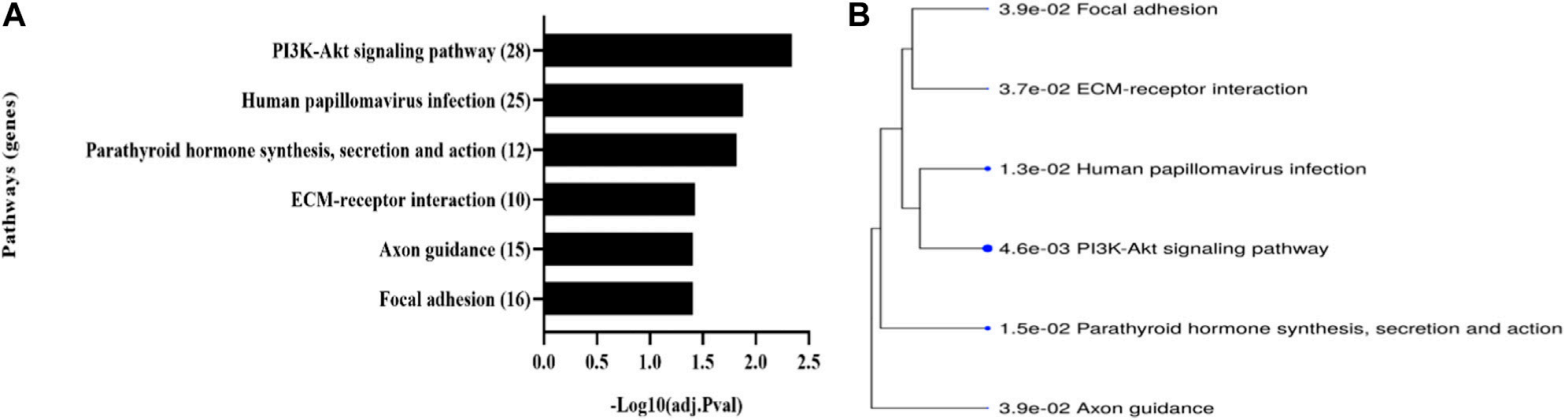
KEGG pathway and gene enrichment. Note: **(A)** KEGG pathway enrichment analysis of hypermethylated genes in the LLM group. The names of KEGG pathways are present on the vertical axis with the number of genes related to them in parentheses. The horizontal axis represents the FDR values in -Log of 10. For this analysis, the *homo sapiens* reference organism was used, considering an FDR <0.05. **(B)** Enrichment analysis tree of hypermethylated genes in the LLM group. The hierarchical clustering tree summarizes the correlation between the significant pathways listed in Figure 2A. Pathways with many shared genes were grouped and larger dots indicate more significant FDR values.

The results showed a total of 979 genes with differently methylated sites between groups (*p* ≤ 0.005; −5% > β > 5%). The sites were hypermethylated in 897 sites and hypomethylated in 82 sites in the LLM group. Figure 3A represents the enrichment of the genes that had hypermethylated sites in the LLM group, demonstrating the FDR values of KEGG pathways (<0.05) and the number of genes related to these pathways. However, we could not find significant relationships for the enrichment of genes with hypomethylated sites in the LLM group.

**FIGURE 4:**
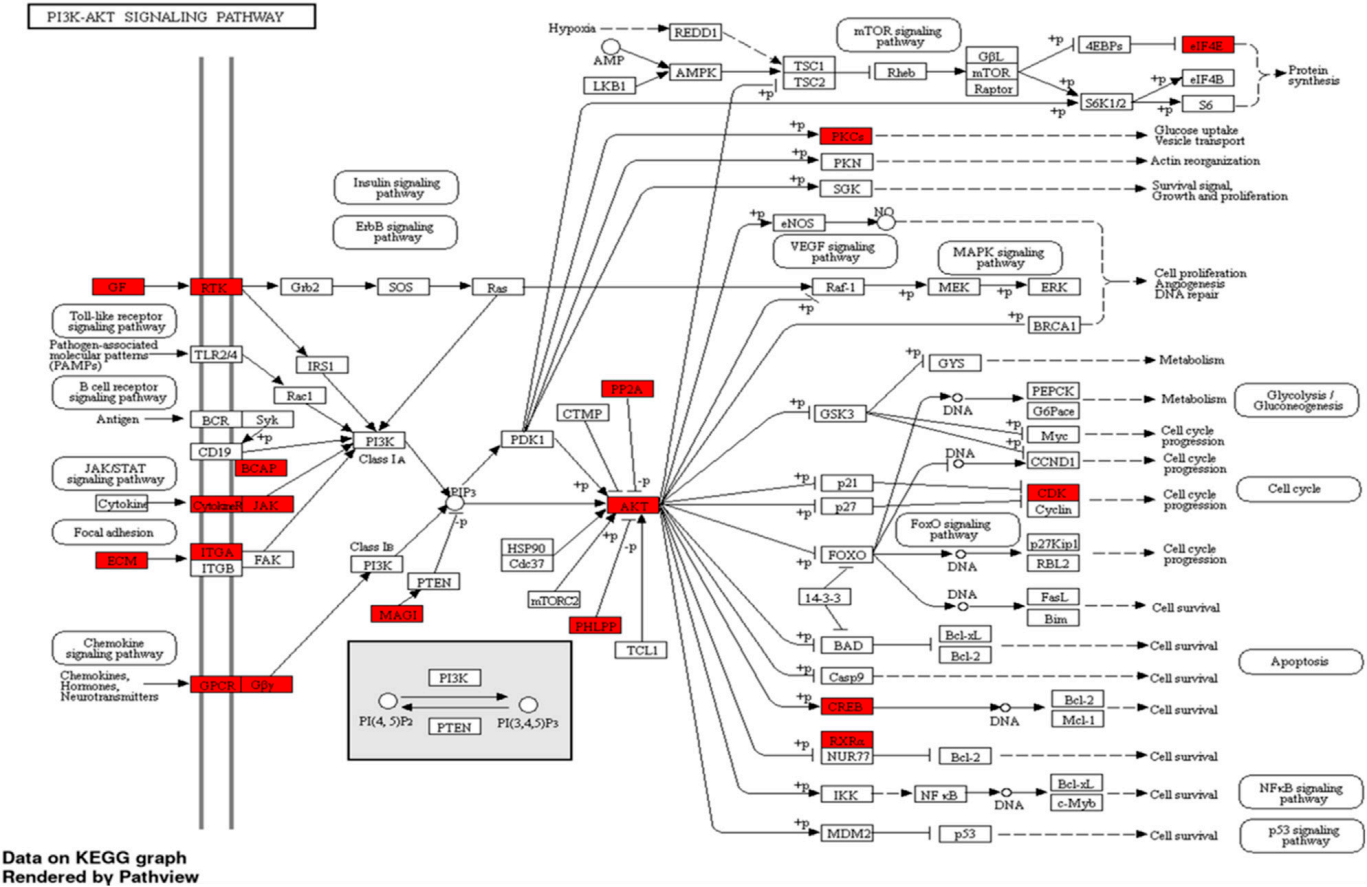
PI3K-Akt KEGG pathway. Note: This image represents the signals and actions of the PI3K-Akt pathway in the human body. The genes highlighted in red showed differences in the methylation profile between the groups.

The KEGG pathways tree in Figure 3B demonstrates the connections between the pathways and the absolute value of the FDR of each one. Thus, it is possible to notice that there is an interaction between these pathways and highlighted the PI3K-Akt pathway with the greatest power of significance (FDR 4.6 × 10^−3^) which is related to the metabolism of lean mass Figure 4.

The analysis of blood tests became relevant following the results of the enrichment of genes that were differently methylated between the HLM and LLM groups since the PI3K-Akt pathway showed a greater significant difference and is also related to the glucose pathway in the human body (Figure 4). Thus, Table 3 presented an analysis of the glucose and insulin profiles in the participants’ blood. However, we did not observe a significant difference (*p* < 0.05) between the groups strengthening the hypothesis that changes in this intracellular signaling pathway are associated with muscle trophism.

**TABLE 3:** Blood glucose and insulin profile in women aged 50–70 years separated by groups (LLM and MLM).

Variables	Less lean Mass (*n* = 20)	More lean Mass (*n* = 20)	*p-value*
Blood Glucose (mg/dL)	104.47 ± 17.85	109.32 ± 28.11	0.53
HA1C (g/dL)	1.05 ± 2.78	0.54 ± 0.44	0.43
HbA1c (%)	5.82 ± 0.70	5.84 ± 0.54	0.94
Insulin (µUI/mL)	10.63 ± 7.96	13.76 ± 10.98	0.32

Legend: Blood Glucose, blood glucose measured after 12 h of fasting; HA1C and HbA1c, these are tests that measure glycated hemoglobin; Insulin, measures blood insulin; g, grams; mg, milligrams; dL, deciliters; ml, milliliters; µUI, micro international unit. We adopted a value of *p* < 0.05 as a significant difference between the groups using the Student’s t-test.

**FIGURE 5:**
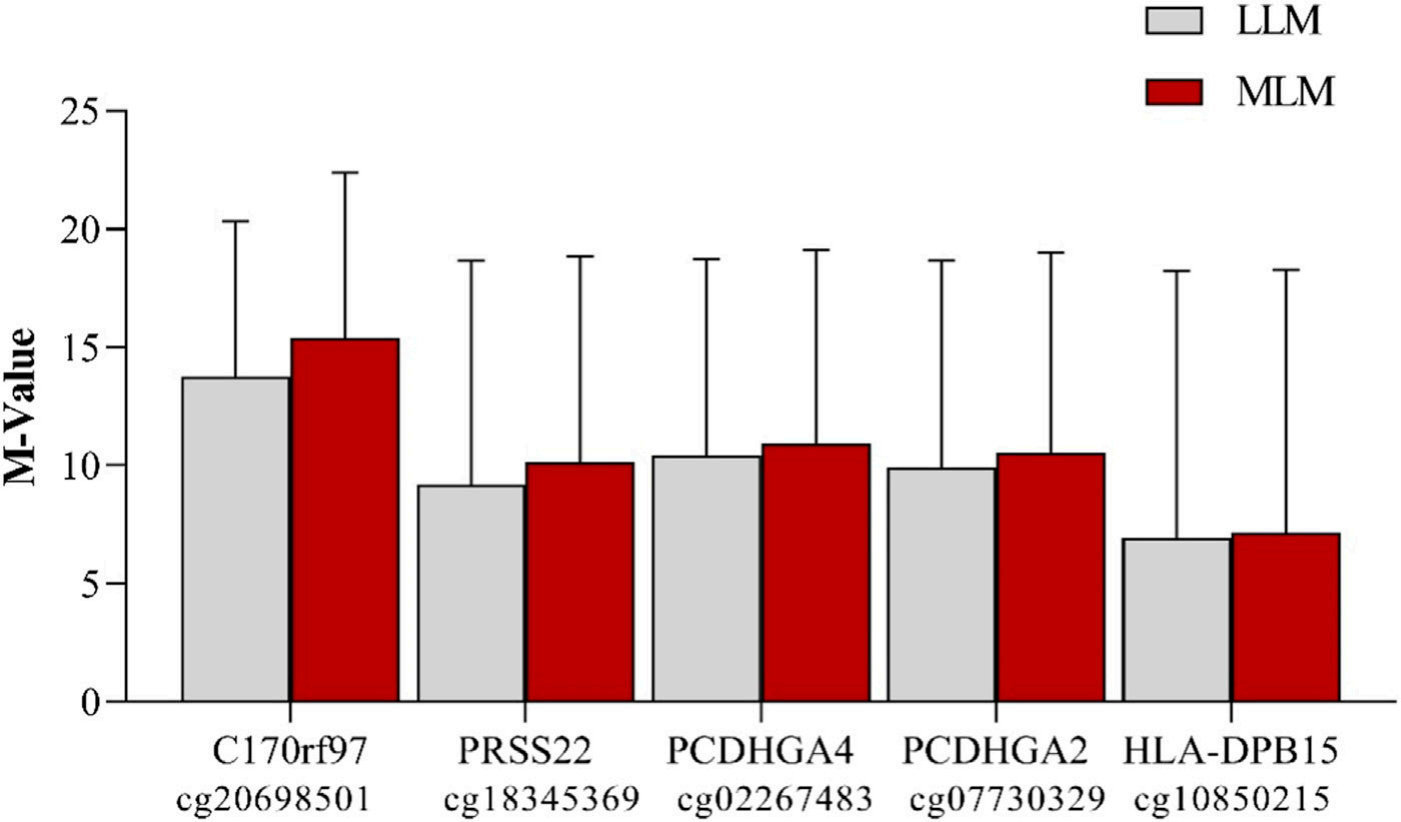
M-value of differently methylated sites between groups and their respective genes derived from DMR analysis. Note: The gene names are on the *X*-axis, and the identification of the sites of each gene is just below them. The sites that were inserted in this group are methylated differently between the groups (*p* ≤ 0.05). Data presented in mean and standard deviation.

DMR analysis revealed that the Bumphunter detected 10 DMRs with a *p*-value ≤0.05. Of the 10 DMRs, one was in the intergenic region, and the other nine were linked to genes. The beta values were transformed into M values, and the sites that showed statistically significant differences between the groups are shown in Figure 5. We found a pattern in the different levels of methylation in the comparisons between groups. The HLM group was hypermethylated in genes in Figure 5. Notably, only the C170rf97 gene was in the TSS1500 promoter zone, and the others were in the body region. The HLA-DPB15 and C170rf97 genes showed differentially methylated sites in the CpG island region, while the others were in the shore region. The M values of the other sites, respective genes, and methylation types are presented in Supplementary File S1.

## 4 Discussion

The current study aimed to analyze the differences in the methylation profile between older women with a higher lean mass and those with a lower lean mass. We observed a significant difference in BMI and lean mass measured by DEXA between HLM and LLM groups. BMI is widely used to estimate adiposity in epidemiological studies as well as clinical practice. However, BMI cannot differentiate fat and lean mass, nor the distribution of body fat (Li and Qi, 2019). On the other side, DXA, although not considered a “gold-standard” method, is an established technique used in clinical and research settings to assess body composition including total and regional LM, and provides useful information regarding clinical and nutritional status (Imboden et al., 2017). The proper assessment and follow-up of obesity and sarcopenia are relevant for the management of the complications of cardiometabolic and musculoskeletal frailty, especially in older adults (Imboden et al., 2017).

One explanation for the differences in body composition between individuals is the link between genetics, environment, and lifestyle, which can facilitate or hinder the improvement of health indicators (Nguyen et al., 1998; Chen et al., 2000; Lee et al., 2011). Interestingly, epigenetics enable the interaction between lifestyle/environmental factors and modifications to DNA and histones, without changes in the DNA sequence. These epigenetic “tags” occurring because of life course events can influence gene expression (Stewart and Sharples, 2022). Thus, our study demonstrated a significant difference in the methylation profile in genes of the PI3K/Akt pathway. The genes related to this pathway had hypermethylated sites in the LLM group compared to the HLM group. These results indicate a possible difference in gene expression between the groups (Baylin and Jones, 2011; Gensous et al., 2019). The literature shows that skeletal muscle hypertrophy can be measured by the induction of insulin-like growth factor-1 (IGF-1), which stands out as a protein growth factor sufficient to induce muscle mass (Coleman et al., 1995; Musarò et al., 2001). According to the literature, IGF-1 acts in part by stimulating the PI3K/Akt pathway, resulting in the activation of targets that induce protein synthesis (Bodine et al., 2001; Rommel et al., 2001). Our data demonstrated that older women with lower values of lean mass have hypermethylated sites in the PI3K-AKT pathway, demonstrating that this pathway could be silenced. Studies demonstrate that Akt activation appears to be sufficient to induce the hypertrophy process *in vivo* (Lai et al., 2004; Mammucari et al., 2007; Izumiya et al., 2008). Acute activation of Akt in a mouse for 2 weeks was sufficient to induce a doubling in skeletal muscle size (Izumiya et al., 2008). Furthermore, signaling between IGF1 and PI3K/Akt may inhibit the effects of myostatin. This protein inhibits myoblast differentiation, blocks the Akt pathway, and deregulates the Akt/mTOR/p70S6 protein synthesis pathway. Thus, myostatin inhibition can cause an increase in skeletal muscle size, stimulating protein differentiation and synthesis (Glass, 2010; Egerman and Glass, 2014).

The PI3K/Akt pathway is also related to glucose and insulin metabolism. This PICK1-activated pathway increases GLUT2 expression and functionally protects pancreatic β cells, promoting insulin secretion and slowing the progression of type 2 diabetes mellitus (Qiu et al., 2020). PI3K/Akt also protects against neural apoptosis and diabetic encephalopathy, which is stimulated by glucose fluctuation (Yan et al., 2020). Ligands such as leptin, insulin, GLP, and growth factors act in the PI3K/Akt pathway and have essential roles in the functioning of this pathway. Among these ligands, insulin is the primary regulator of this pathway. Insulin, when it is secreted after a meal, promotes an increase in glucose utilization and a reduction in gluconeogenesis in the liver and muscles; increased deposition of lipids in the body; reduced circulation of free fatty acids in adipose tissue; reduced appetite in the brain; and increased production of insulin in the pancreas; as well as regulating the balance of lipid and glucose metabolism by stimulating the functioning of the PI3K/Akt pathway. However, excess energy, as in obesity, can impair the signaling of this pathway, causing insulin resistance (Huang et al., 2018).

We also found in the HLM group, hypermethylated sites in genes related to extracellular matrix (ECM) receptor interaction, axon orientation, and focal adhesion. The ECM interaction pathway is essential for regulating intercellular interaction and tissue architecture. The ECM undergoes considerable remodeling in situations of injury, repair, short-term overeating, metabolic dysfunction, and obesity (Tam et al., 2014; Tam et al., 2017; Ahmad et al., 2018; Ahmad et al., 2020). Studies demonstrate that the ECM pathway can increase biochemical signals, stimulating myogenesis. In addition, it is related to the regulation of insulin signaling in cells (Lin et al., 2009; Waite and Eickholt, 2010; Alan et al., 2013; Tam et al., 2014; Tam et al., 2017; Ahmad et al., 2018; Ahmad et al., 2020). When insulin is bound to cell membrane receptors, signal transduction pathways activate PI3K-Akt and insulin receptor substrate (IRS) −1, which act on glucose uptake through GLUT4. Therefore, low expression of the genes of the ECM interaction pathway can result in impairment in post-stimulus insulin signaling, which can lead to the development of insulin resistance and type 2 diabetes mellitus (Lin et al., 2009; Waite and Eickholt, 2010; Alan et al., 2013; Tam et al., 2014; Tam et al., 2017; Ahmad et al., 2018; Ahmad et al., 2020). The axon guidance (OA) pathway is related to the connectivity and repair of neurons throughout life and guides the brain’s wiring during fetal development. The neuron signaling pathway complex and its intracellular signaling is related to various cellular responses: trafficking events, gene transcription, cytoskeletal remodeling, ubiquitination, and protein translation (Lin et al., 2009; Waite and Eickholt, 2010; Alan et al., 2013). The PI3K pathway assists the OA pathway in controlling neuronal migration, neuronal morphogenesis, and the development of dendrites and synapses. These pathways are essential in stages of brain development, which include mediating extrinsic and intrinsic signals, such as growth factors, axon guidance, extracellular matrix components, and signaling components that act in translation. However, the literature does not explicitly state a direct relationship between this pathway and lean mass or glucose metabolism (Waite and Eickholt, 2010; Alan et al., 2013). Focal adhesions (FA) are sites where there is adhesion mediated by integrin and proteoglycan bound to the actin cytoskeleton. These sites are modified by cells in response to alterations in molecular composition, structure, and physical forces present in the extracellular matrix. FA regulates integrin signaling and function complexes, physical and biochemical cellular behaviors, cell proliferation, survival, migration, and invasion (Wozniak et al., 2004). This pathway is also related to insulin signaling, glycogen synthesis, and differentiation in muscle cells. Some studies have shown that PA promotes the architectural remodeling of skeletal muscle in response to mechanical stimuli, such as what occurs during physical exercise (Crossland et al., 2013; Graham et al., 2015; Franchi et al., 2018; Luca et al., 2020). In addition to the PI3/Akt, the FA pathway can act against insulin resistance (Bisht et al., 2008; Gupta and Dey, 2009). It is possible to notice that all these pathways are interconnected with the PI3K/Akt pathway. However, nothing in the literature relates all these pathways to lean mass or glucose metabolism.

From the results of our work, it is not possible to state differences in the expression of genes. However, in our study, the absence of significance between groups in blood glucose and muscle functionality (*p* < 0.05) indicated that differences in lean mass were the determining factor in differentiating the methylation profile of the genes of the PI3K/Akt pathway between the groups. In addition, this exploratory study points to the need for case-control studies to analyze potential associations of the pathways with the PI3K/Akt pathway and the control of muscle trophism.

The DMR analysis detected five regions of specific genes that presented sites with statistical differences between the groups for M-values. The areas of these genes had a different methylation profile according to the group (LLM and HLM). The expression of the protocadherin gamma gene group (PCDHGA4 and PCDHGA2) is negatively correlated with muscle strength (Huang et al., 2018). In addition, it may be related to muscle denervation and reinnervation (Huang et al., 2018). Although no studies demonstrate the relationship between the expression of PRSS22, C170rf97, and HLA-DPB15 genes and lean mass, studies show variations in the human leukocyte antigen (HLA) gene polymorphism are related to sarcopenia. In parallel, the expression of these genes is associated with impaired glucose metabolism (Solini et al., 2010; Jones et al., 2020). It is worth mentioning as a limitation that both groups were pre-diabetic, which can change the methylation profile and lean muscle mass of the participants. Studies that compare participants’ profiles of glucose metabolism within the normal range are necessary. Another limitation could be the sample size. Nevertheless, although the sample size is small, the associations found between methylation changes and body composition were relevant considering this study as a hypothesis-generating study. Thus, larger confirmatory study is needed in the field of epigenetics and body composition.

In summary, our study demonstrated significant differences in the methylation profiles between women aged 50–70 years who have a higher lean mass and with a lower lean mass in the context of aging. The PI3K/Akt pathway had the lowest FDR value, and all other pathways are related to it, suggesting that this pathway may be the most affected by lean mass composition. Our results demonstrate a differentiation between specific sites of different genes, which have essential functions in body composition and energy metabolism, supporting future studies that aim to relate lean mass with epigenetics.

## Data Availability

The datasets presented in this study can be found in online repositories. The names of the repository/repositories and accession number can be found below: GEO, GSE199700.
